# X-Ray Spectrometric Analysis of Noble Metal Dental Alloys

**DOI:** 10.6028/jres.068A.002

**Published:** 1964-02-01

**Authors:** B. W. Mulligan, H. J. Caul, S. D. Rasberry, B. F. Scribner

## Abstract

The analysis of noble metal dental alloys for the various constituent elements is a difficult and tedious task by chemical or fire assay procedures. X-ray spectroscopy offered the possibility of increased speed, especially if solid metal samples were employed. This technique was investigated particularly with respect to the analysis of dental alloys having the nominal composition in percent, of gold 72, silver 12, copper 10, platinum 2, palladium 2, and zinc 2. Alloys were prepared by melting the component elements in a high frequency furnace and casting the metal into disk form. Compositions of the castings were determined by chemical analysis. Optimum procedures for casting the sample and for X-ray analysis were established, and analytical curves were developed relating concentrations to measured intensities of the X-ray lines Au L*_β_*, Ag K*_α_*, Cu K*_α_*, Pt *L_α_*, Pd K*_α_*, and Zn K*_α_*. The observed typical coefficients of variation for the method were Au 0.34 percent, Ag 0.44 percent, Cu 2.2 percent, Pt 1.6 percent, Pd 1.2 percent, and Zn 0.72 percent. The results indicate that the method is sufficiently accurate and has marked advantages of speed and simplicity compared to chemical analysis.

## 1. Introduction

The analysis of noble metal alloys by traditional methods of solution chemistry [3]^2^ is one of the more complex and time-consuming tasks confronting the analytical chemist. Three to four man-weeks are usually required for the analysis of three samples in duplicate. The methods involve painstaking separations with numerous precipitations and filtrations.

Fire assay techniques also are used but chiefly for the determination of gold. If platinum and palladium are present in the sample being assayed, the alloy has a much higher melting point and there are more losses due to volatilization [2]. In addition, further chemical separations are necessary to remove the platinum and palladium before gravimetrically determining the gold. In the assay technique the base metals are either absorbed into the cupel or volatilized and hence are not determined.

In conjunction with the problem of analysis of gold alloys for the Research Group of the American Dental Association, the development and evaluation of an instrumental method of analysis was considered. X-ray spectrometry with its high precision offered the best possibility for a rapid method, especially if wet chemical processes could be eliminated in preparing the samples. A typical composition for the alloys to be analyzed was, in percent, gold 72, silver 12, copper 10, platinum 2, palladium 2, and zinc 2.

There are only a few references in the literature concerning the X-ray spectrometric determination of the noble metals. MacNevin and Hakkila [6] made determinations of palladium, platinum, rhodium, and iridium in a liquid sample that was applied to heavy chromatographic paper, dried, and compared with a previously calibrated color chart. Bacon and Popoff [1] described quantitative X-ray fluorescence analyses of glass for 15 or more elements including platinum and silver. Other references of interest include the determination of platinum in a petroleum reforming catalyst [4], and the determination of silver in photographic films [5].

## 2. Experimental Procedure

In order to investigate the direct X-ray spectrometric analysis of solid alloys, samples were synthesized initially by melting together in a high frequency furnace solid metal pieces of the desired component elements. Four samples were prepared in this manner.

The molten alloys of these elements were cast in the shape of disks approximately ⅛-in. thick with a diameter of 1¼ in. to fit the sample holders of the X-ray spectrometer to be employed. Each disk so cast weighed about 35 g. The surfaces of the disks were then precision machined for the X-ray measurements. The compositions of the castings were determined by wet chemical analysis according to the method of Gilchrist [3], and the results of the analysis are shown in table 1.

**Figure 1:**
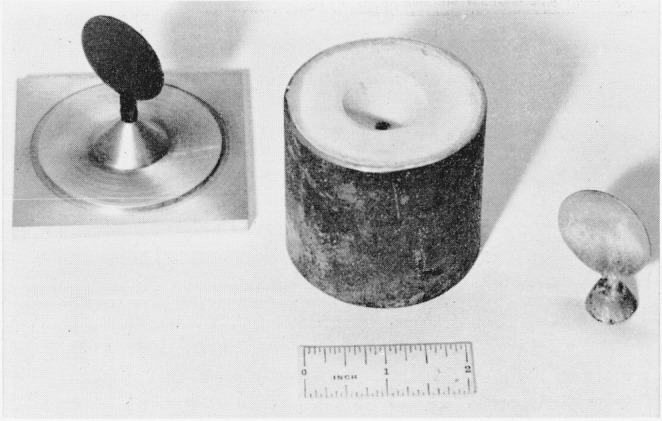
Preparation of sample—(left) Wax disk mounted on metal cone; (center) metal cylinder (inverted) containing mold cavity of wax disk; (right) noble metal alloy disk before removal of sprue.

**Figure 2:**
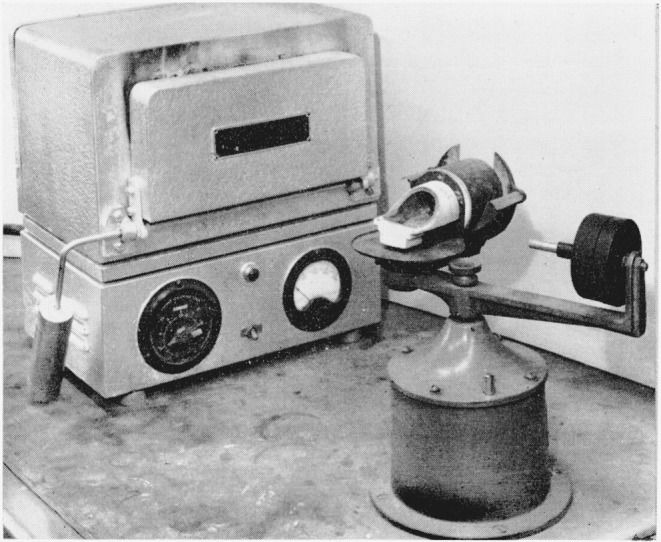
Preparation of sample—furnace and centrifugal casting machine with mounted mold cylinder.

The optimum conditions for X-ray fluorescence intensity measurements were determined experimentally and are given in table 2. Using these parameters, measurements of intensities at the X-ray wavelengths listed were made on the disks for all six constituent elements. The resultant analytical curves were found generally to be linear and the precision of repeated determinations appeared favorable for an analytical method. Before carrying the development of the method further, it was considered advisable to establish a preparation technique requiring a smaller quantity of precious metal per sample. The technique generally applied to prepare castings in dentistry, known as investment casting or the “lost wax” process [8, 9], appeared promising. This technique was applied, using a small centrifugal casting machine, to obtain disks of the gold alloys varying in thickness from ⅛ to 164 in. The various steps of this process are illustrated in figures 1 and 2. Disks are cut from wax sheets of the desired thickness and mounted vertically on edge on the tip of a brass cone as shown in figure 1. A metal cylinder is then placed around the disk and cone and the cylinder is filled with an investment material (consisting of 50% crystobolite and 50% gypsum). The investment hardens in a few minutes and the filled cylinder is placed in a furnace and heated to approximately 700° C. During the heating the wax volatilizes completely leaving a mold cavity having the dimensions of the wax disk.

The heated cylinder is removed from the furnace, cooled to about 500 °C, and mounted on the centrifugal casting machine shown in figure 2. In the machine the mold is shown adjacent to the small refractory melting cup at the center. The gold alloy is melted in the cup by means of a torch and is cast immediately by releasing the spring loaded arm of the machine. The molten metal is forced into the mold cavity where it solidifies in disk form. The furnace in the background served for preparing the mold.

After cooling, the mold is broken apart and the metal disk and sprue are extracted. The sprue is cut from the disk by means of a jeweler’s saw, and the metal disk sample is mounted in methyl methacrylate resin for handling during surface preparation and analysis. The exposed surface of the resin- mounted sample is prepared for analysis by wet grinding to a flat surface, finishing with a 600-grit abrasive.

X-ray intensity measurements for the selected wavelengths were made for all of the disks, and no differences could be detected within the precision of the determinations among the disks of the same alloy having different thicknesses. Subsequently, disks of 132-in. thickness were selected as the best compromise between rigidity of sample and minimum amount of alloy per sample. The disk and sprue together weighed about 20 g with the sprue accounting for slightly over half of this weight.

Samples of the four previously analyzed gold alloys were prepared in duplicate by this technique and X-ray fluorescence measurements were made in the spectrometer in a sequence designed to minimize effects of instrumental drift [7]. The overall time required for this procedure was approximately 5 hr, which included about 3 hr for sample preparation and about 2 hr for X-ray measurements. The effect of melting and casting on the apparent composition of the alloys will be discussed later in the text.

## 3. Results and Discussion

**Figure 3:**
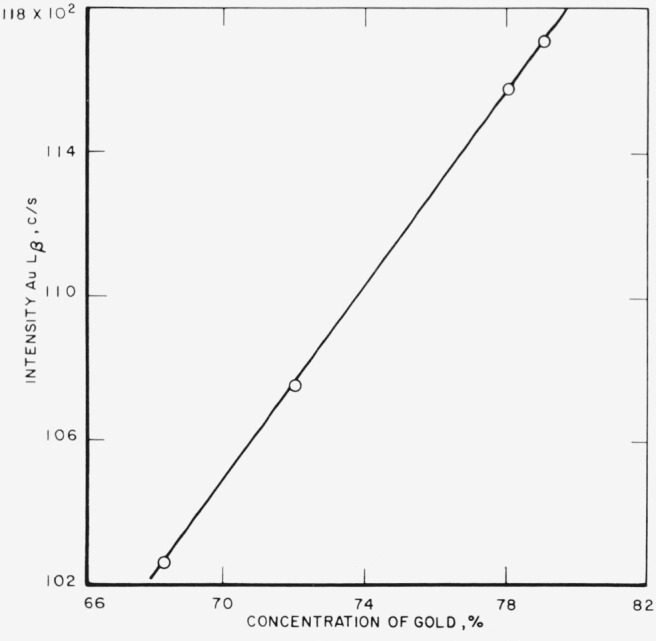
Analytical curve for gold.

The analytical curves obtained from these runs for gold, silver, copper, platinum, palladium, and zinc are shown in figures 3 through 8. Each point on a curve is the mean of four individual determinations. The precision of the method, calculated as the coefficient of variation for an individual determination in a group of four, is given in table 3. [Fig f4-jresv68an1p5_a1b] [Fig f5-jresv68an1p5_a1b] [Fig f6-jresv68an1p5_a1b] [Fig f7-jresv68an1p5_a1b] [Fig f8-jresv68an1p5_a1b]

In the case of platinum, sample number two resulted in a higher count compared to the linear relationship of the other three samples for reasons as yet unknown. A least squares treatment of the data indicated that the point was in error by 0.14 percent.

The theoretical precision that should be obtained solely on the basis of the counting statistics is equal to the reciprocal of the square root of the total number of counts per measurement. In the case of gold, silver, and copper, where 256,000 counts were measured per determination, this would be about 0.20 percent. For platinum, palladium, and zinc, where 128,000 counts were measured, it would be about 0.28 percent. This precision was approached in the case of gold and silver, but not in the other cases, possibly because of the greater influence of background variation (which could not be measured simultaneously) when the line-to- background ratio is low.

A possible drawback to this procedure is the loss of more volatile constituents in the melting and casting operation. To investigate this possibility, samples were repeatedly melted and cast in an automatic machine which cast the metal when a selected temperature was reached. The samples were melted, then heated to 1080 °C and cooled to 870 °C six times before casting. The castings were then analyzed by the X-ray procedure described and compared with samples of the same material melted once at 1080 °C and cast immediately. Within the precision of the method no differences could be detected between the samples prepared by the two casting procedures, indicating negligible loss on melting.

The spectrometer used (table 2) is a researchtype instrument in which individual determinations must be made sequentially (since it has only one crystal and detector) and wavelength settings must be changed manually. More automatic multichannel instruments are available which would decrease the measurement time by a substantial factor.

The results indicate that the method is sufficiently precise and has distinct advantages of simplicity of sample preparation and speed of measurement compared to chemical analysis. The melting and casting procedure employed in this work would appear to offer advantages as a general method for preparing samples and standards especially for X-ray spectrometry. The casting procedure probably could be speeded up by use of a permanent split mold instead of the investment mold. The extension of the technique to the analysis of silver alloys is currently in progress.

## Figures and Tables

**Figure 4 f4-jresv68an1p5_a1b:**
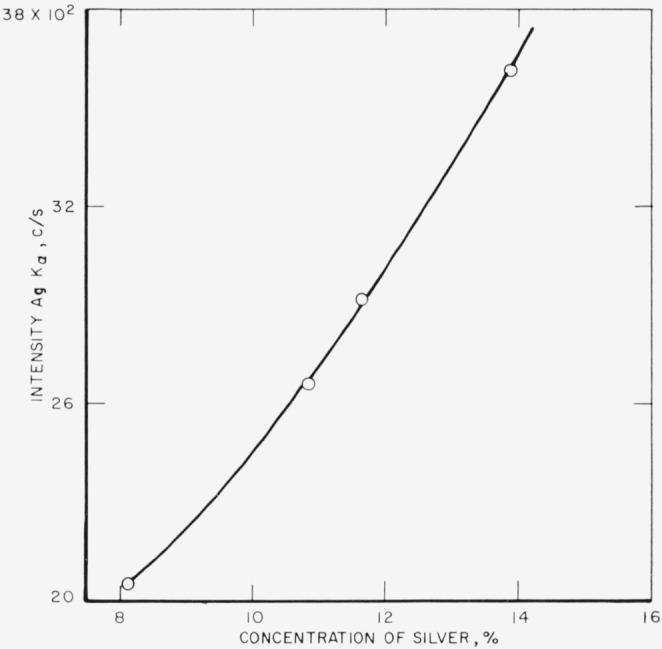
Analytical curve for silver.

**Figure 5 f5-jresv68an1p5_a1b:**
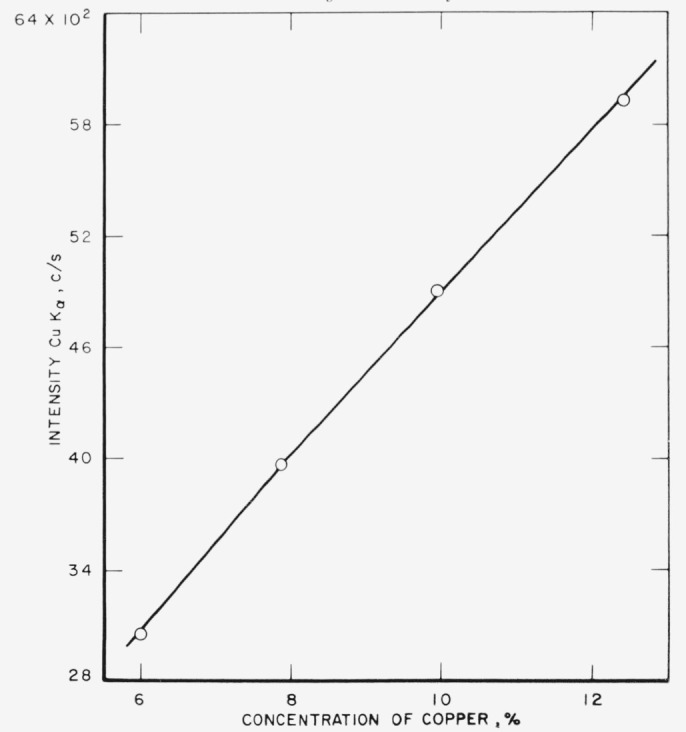
Analytical curve for copper.

**Figure 6 f6-jresv68an1p5_a1b:**
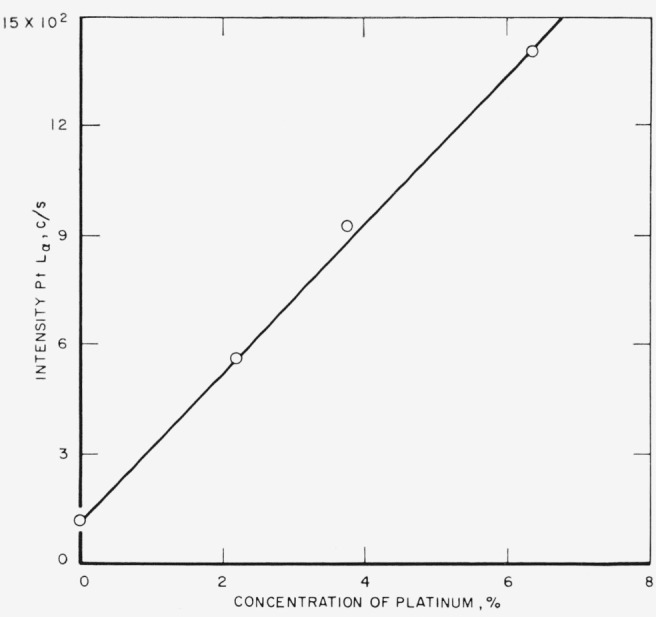
Analytical curve for platinum.

**Figure 7 f7-jresv68an1p5_a1b:**
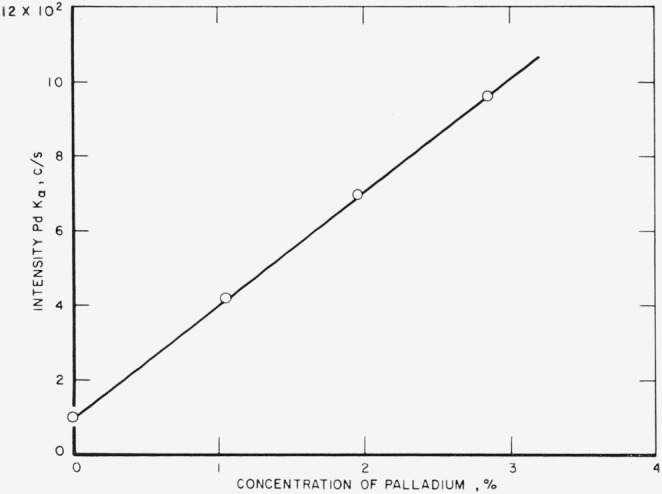
Analytical curve for palladium.

**Figure 8 f8-jresv68an1p5_a1b:**
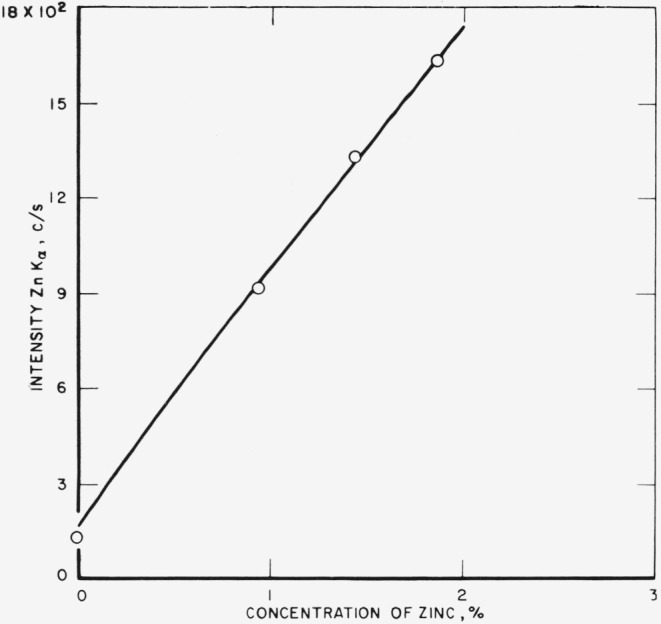
Analytical curve for zinc.

**Table 1:** Results of chemical analysis

Element	Alloy No.			
	1	2	3	4
				
	*Percent*	*Percent*	*Percent*	*Percent*
Au	68.27	72.01	78.08	79.08
Ag	11.63	10.86	8.13	13.88
Cu	11.88	9.95	7.86	6.01
Pt	6.34	3.76	2.17	.00
Pd	.00	1.97	2.84	1.04
Zn	1.87	1.44	.94	.00
Totals	99.99	99.99	100.02	100.02

**Table 2:** Operating parameters 1 for noble metal alloy analysis

Element	X-ray line	Degrees, 2*θ* LiF crystal	Wavelength X-ray tube			Counts per determination
				
			*Å*	*kv*	*ma*	
Au	L*_β_*	31.19	1.083	50	30	256,000
Ag	K*_α_*	15.95	0.559	50	45	256, 000
Cu	K*_α_*	44.96	1.540	50	45	256,000
Pt	L*_α_*	38.05	1.313	50	45	128,000
Pd	K*_α_*	16.70	.585	50	45	128,000
Zn	K*_α_*	41.74	1.435	50	45	128,000

1 Norelco Inverted-Sample Three-Position Spectrograph with molybdenum target X-ray tube, lithium fluoride crystal, and scintillation counter detector (Philips Electronic Instruments, Mt. Vernon, N.Y.).

**Table 3:** Reproducibility of single determinations

Au concentration	CV^1^	Ag concentration	CV	Cu concentration	CV
					
%		%		%	
68.27	0.43	11.63	0.17	11.88	2.07
72.01	.23	10.86	.66	9.95	2.32
78.08	.41	8.13	.30	7.86	2.60
79.08	.30	13.88	.63	6.01	1.74
					
Pt concentration	CV	Pd concentration	CV	Zn concentration	CV
					
%		%		%	
6.34	1.15	0.00	2.24	1.87	0.73
3.76	1.63	1.97	1.32	1.44	.81
2.17	2.64	2.84	1.18	0.94	.62
20.00	1.36	1.04	1.19	.00	2.15

1 CV=Observed coefficient of variation for an individual determination in a group of four $\text{CV}=\frac{100}{\bar{c}}\sqrt{\frac{{\left(\Sigma c-\bar{c}\right)}^{2}\cdot }{n-1}}$

2 Coefficient of variation for zero concentration is the value for the background radiation.

## Footnotes

2 Figures in brackets indicate the literature references at the end of this paper.
